# MD Codes™: A Methodological Approach to Facial Aesthetic Treatment with Injectable Hyaluronic Acid Fillers

**DOI:** 10.1007/s00266-020-01762-7

**Published:** 2020-05-22

**Authors:** Maurício de Maio

**Affiliations:** MD Codes Institute, Rua Santa Justina, 660 - cjs 121 e 124, Via Olímpia, Sao Paulo, SP Brazil

**Keywords:** Aesthetics, Dermal fillers, Hyaluronic acid, Rejuvenation, Skin aging, Skin wrinkling

## Abstract

**Background:**

Patients often seek aesthetic correction of facial deficiencies (e.g., lines and folds) that are rarely the underlying cause of dissatisfaction with their appearance. Use of a more holistic approach focused on improving the emotional messages of the face (e.g., looking less sad) may improve patient satisfaction with treatment outcomes. The MD Codes™ system was developed to increase clinician success rates by reducing variability in the technical aspects of hyaluronic acid (HA) filler treatment and focusing on addressing unfavorable emotional attributes of the face.

**Methods:**

The MD Codes, or medical codes, represent specific anatomical subunits for injection of HA fillers. Each MD Code includes information regarding the target depth of injection, the proper delivery tool (needle or cannula) and delivery technique (e.g., aliquot, bolus, fanning), and the minimum product volume recommended to achieve visible, reproducible results (active number). During treatment planning, the appropriate MD Codes are selected using algorithms focused on lessening unfavorable facial attributes (a saggy, tired, sad, or angry look) and enhancing positive attributes (an attractive, younger, more contoured, or feminine [soft] or masculine look).

**Results:**

Three case studies are presented to illustrate how the MD Codes and their algorithms were used to address sagginess, tiredness, and sadness in two women and one man.

**Conclusions:**

MD Codes provide a universal symbolic language for reducing variability in injection technique. The platform provides user-friendly algorithms to help clinicians increase patient satisfaction by going beyond treatment of lines and folds and to focus on reducing unfavorable facial attributes.

**Level of Evidence IV:**

This journal requires that authors assign a level of evidence to each article. For a full description of these Evidence-Based Medicine ratings, please refer to the Table of Contents or the online Instructions to Authors www.springer.com/00266.

**Electronic supplementary material:**

The online version of this article (10.1007/s00266-020-01762-7) contains supplementary material, which is available to authorized users.

## Introduction

Patients who undergo facial aesthetic procedures may be dissatisfied with treatment outcomes [1–3]. They often focus on particular areas with which they are unhappy, such as the periorbital area or jowls, and ask the clinician providing treatment to specifically address those features [4–8]. Even as patients believe that the objective of treatment with hyaluronic acid (HA) fillers is to simply eliminate distracting lines and folds, they may be dissatisfied with treatment because they were expecting improvements beyond the elimination of isolated flaws. Patients commonly hope for more global improvement, expecting to achieve a more cheerful, more relaxed, or less tired look after treatment [9].

Faces can convey a variety of emotional cues or messages that often do not reflect a patient’s true feelings. For example, a patient’s face may look tired when the patient is not feeling tired or may convey sadness when the patient is not feeling sad [9–14]. Studies have demonstrated that negative emotional messages are associated with specific facial deficiencies [9–11, 13, 15]; for example, an angry appearance may be caused by glabellar lines or a tired look caused by eye bags [10, 11, 13, 15]. Changes that occur with aging in the skin, soft tissue, and bones of the face and cause such deficiencies may result in the accumulation of these negative emotional messages [9, 11, 13]. However, treatment of only one isolated area (e.g., the eye bags) may not lead to a successful aesthetic outcome.

Several authors have suggested that rather than treating individual facial deficiencies, clinicians providing facial aesthetic treatment should address the emotional messages or miscues of the patient’s face [9, 10, 15]. In this author’s experience, patient satisfaction with treatment is improved when treatment focuses on reducing unfavorable facial messages and on increasing favorable attributes, rather than treating isolated areas. A number of emotional cues are described in the literature, including anger, fear, fatigue, sadness, and happiness [9–13, 15], In this paper, facial messages are grouped as four unfavorable attributes (looking tired, looking sad, having a saggy appearance, and having an angry look) and four favorable or positive attributes (looking attractive, younger, more contoured, and either feminine [soft] for women or masculine for men).

Addressing unfavorable facial attributes is challenging given the many variables that influence treatment success, defined here as a reduction in negative attributes and enhancement of positive attributes (Table 1). Some variables, such as a patient’s age, gender, and ethnicity, are not within the clinician’s control. Each of those characteristics in turn can independently affect fat content, muscle activity, and skin quality and laxity, resulting in an infinite variety of faces. However, the technical aspects of treatment, such as the product used and injection techniques applied, can be more precisely controlled. The MD Codes™ (or medical codes) is a system developed by the author to provide specific injection guidelines giving the precise location, layer, tool, delivery system, and product volume information to be used to achieve optimal results, regardless of patient age, gender, or ethnicity. The achievement of successful results, defined here as the reduction in negative or unfavorable attributes and enhancement of positive attributes, will vary between clinicians of different skill levels and experience; however, the MD Codes guidelines can improve the performance of the novice clinician, while also theoretically enhancing the success rate of more experienced clinicians. The use of the MD Codes to address unfavorable emotional messages of the face has been presented online and in seminars worldwide. While materials describing the MD Codes have been provided in conjunction with those seminars [16, 17], this article provides the first peer-reviewed description of the MD Codes system and its algorithms. Case studies are presented to illustrate its use.

**Table 1:** Key sources of variability in minimally invasive aesthetic treatment outcomes

Variable	Examples
Patient	Age Ethnicity Gender Physiology Bone structure Fat content Muscle activity Skin quality
Product	Type of filler (biodegradable and nonbiodegradable) HA brand technology Concentration Degree of cross-linking
Technique	Injection details Location Unit Subunit Layer Mucosa, dermis, sub-dermis, subcutaneous, fat pads, muscle, bone Volume
	Injection tool Needle type and gauge Cannula type and gauge
	Injection delivery Micro-aliquot Aliquot Bolus Linear Fanning
Clinician	Level of technical skill Years of experience Depth of knowledge of facial anatomy Breadth of experience in the patient population (e.g., by ethnicity, gender)

## Symbolic Language of the MD Codes

**Fig. 1:**
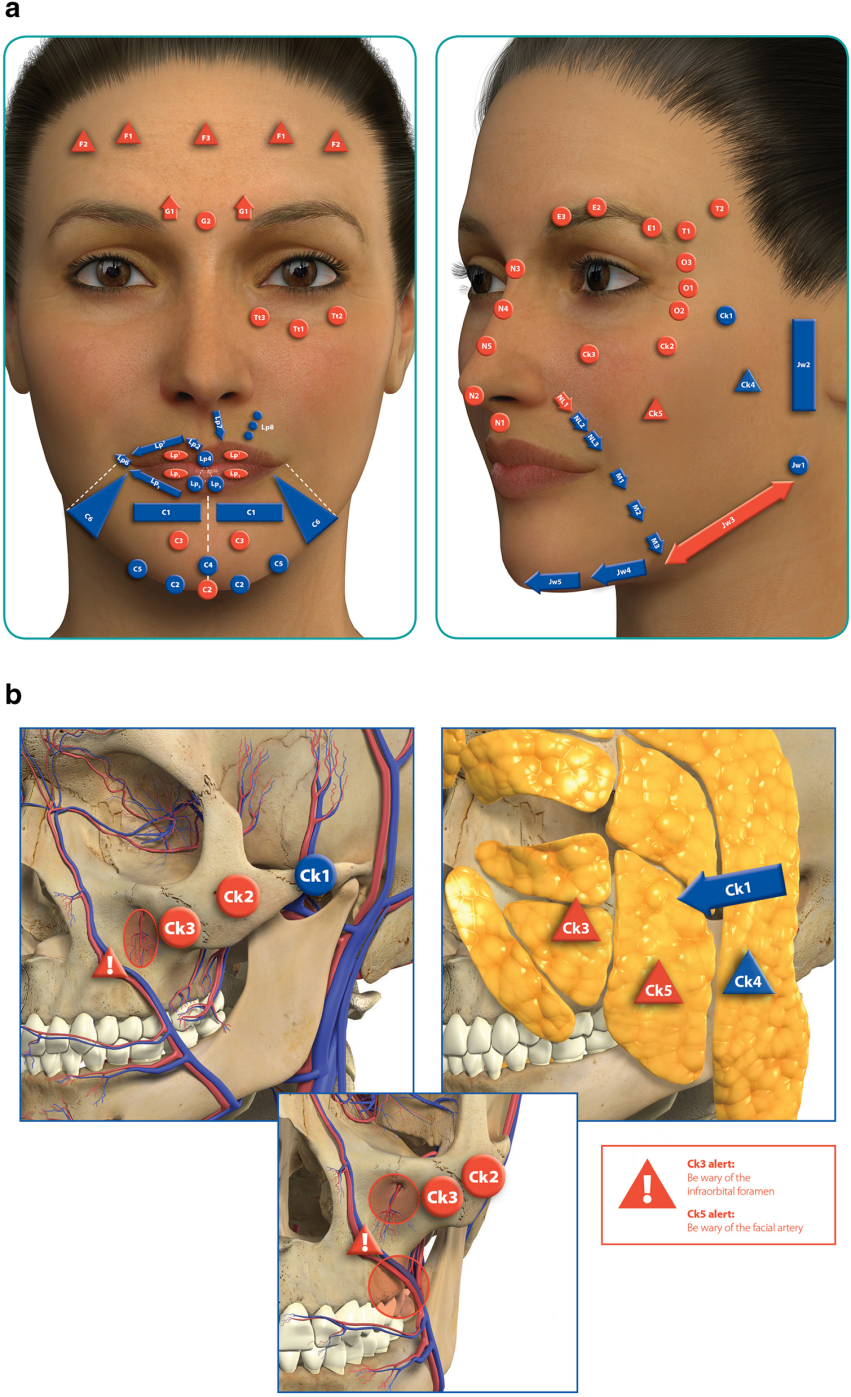
MD Codes anatomical correlates (**a**) and in relation to the topographical anatomy of the cheek (**b**). Red codes denote alert areas. Reprinted with permission from Allergan plc, Dublin, Ireland

The MD Codes are letters, numbers, shapes, and colors (Table 2; Fig. 1) representing precise anatomical sites and procedures for the injection of HA fillers that may be understood in any language and that serve as a platform of communication between clinicians of all skill levels. Injection sites are described using a combination of letters and numbers; the letters signify anatomical units (e.g., the cheek, temple, or chin) and the numbers signify subunits, such that each code indicates a single, precise injection site (Table 3; Table S1). For example, the cheek is represented by the letters Ck, and subunits of the cheek are numbered: Ck1 = zygomatic arch, Ck2 = zygomatic eminence, Ck3 = anteromedial cheek–midcheek, and so on. The MD Codes numbers do not reflect the sequence in which the injections should be administered, but instead provide a checklist of items that the clinician can mark when assessing each facial unit. MD Codes in red denote alert areas, where there are sensitive structures, such as neurovascular bundles in facial danger zones [18]. These red codes (Fig. 1) remind the clinician to be cautious in these areas when using needles and to consider the use of cannulas instead. The alert codes should never be used to guide injection by novice clinicians. As discussed below, treatment of alert areas should only be delivered by highly trained experts with extensive injection experience, thorough knowledge of the anatomy and physiology of each area, and the ability to manage severe complications, should they occur.

**Table 2:** Summary of the components of the MD Codes

Component	Meaning
Letter	The anatomical area (e.g., Ck = cheek)
Number	The subunits of the anatomical unit (e.g., Ck1 = zygomatic arch; Ck2 = zygomatic eminence)
Number location	The side of the face (e.g., Ck1 r = the zygomatic arch on the right side; Ck1 l = the zygomatic arch on the left side)
Number position	Superscript (X^n^) refers to upper areas (e.g., Lp^1^ = vermilion body of the upper lip) Subscript (X_n_) refers to lower areas (e.g., Lp_1_ = vermilion body of the lower lip)
Color	Red color denotes alert areas, and additional caution must be taken if injecting at or near these sites, for patient safety
Shape	Technical delivery of the product (e.g., = needle, = cannula, = fanning, = aliquots; = bolus)

**Table 3:** MD codes and injection guidance for anatomical sites using hyaluronic acid fillers

Anatomical unit MD code	Injection area	Target depth of injection	Injection device	Injection delivery	Alerts	Active number per side (mL)^a^
*Foundation*						
Cheek (*Ck*)						
*Ck1*	Zygomatic arch	Supraperiosteal^b^	Needle			0.1 + 0.1 + 0.1^c^
*Ck1 TML*	Zygomatic arch	Sub-SMAS	Cannula			0.5
*Ck2*	Zygomatic eminence	Supraperiosteal^b^	Needle		Be wary of the zygomaticofacial artery^d^	0.2
*Ck3*	Anteromedial cheek	Supraperiosteal^b^	Needle		Be wary of the infraorbital artery^d^	0.3
		Deep malar fat pad	Cannula			0.5
		SOOF	Cannula			0.5
*Ck4*	Lateral lower cheek/parotid area	Subcutaneous	Cannula		Be wary of the parotid gland	0.5
*Ck5*	Submalar/buccal area	Subcutaneous	Cannula		Be wary of the buccal nerve, facial vein and artery	0.5
*Contour*						
Upper face						
Temple (*T*)						
*T1*	Anterior temple	Supraperiosteal^b^	Needle		Be wary of the superficial frontal artery and the deep temporal arteries^d^	0.5
*T2*	Posterior temple	Supraperiosteal^b^	Needle		Be wary of the superficial frontal artery and deep temporal arteries^d^	0.5
*T1/T2*	Anterior and posterior temple	Temporal fascia	Cannula			0.5
Lower face						
Chin (*C*)						
*C1*	Labiomental angle	Subcutaneous	Cannula			0.5^e^
*C2*	Chin apex	Subcutaneous	Cannula			0.3
	Chin apex	Supraperiosteal^b^	Needle^d^			0.3 (only midline)
*C3*	Anterior chin	Supraperiosteal^b^	Needle^d^		Do not go too lateral due to mental artery^d^	0.3
*C4*	Anterior chin/soft tissue pogonion	Subcutaneous	Needle			0.3 (only midline)
*C5*^*f*^	Lateral lower chin	Supraperiosteal^b^	Needle^d^			0.3
*C6*	Lateral chin	Subcutaneous	Cannula			0.5
Jowls *(Jw)*						
*Jw1*	Mandible angle	Supraperiosteal^b^	Needle^d^			0.5
*Jw1*	Mandible angle	Subcutaneous	Cannula			0.5
*Jw2*	Pre-auricular area	Subcutaneous	Cannula		Be wary of parotid gland	0.5
*Jw3*	Mandible body	Subcutaneous	Cannula		Be wary of the superficial temporal artery and the parotid gland	1.0
*Jw4*	Lower prejowl	Subcutaneous	Cannula			0.5
*Jw5*	Lower anterior chin	Subcutaneous	Cannula			0.5
*Refinement*						
Periorbital						
Forehead (*F*)^g,h^					Injection should be avoided in the 2-cm region above the orbit	
*F1*	Medial forehead	Supraperiosteal^b^	Cannula		Be wary of the supraorbital artery	0.5
*F2*	Lateral forehead	Supraperiosteal^b^	Cannula		Be wary of the superficial temporal artery	0.5
*F3*	Central forehead	Supraperiosteal^b^	Cannula		Be wary of the supratrochlear artery	0.5
Lateral orbital (*O*)						
*O1*	Central lateral orbital	Supraperiosteal^b^	Cannula	Figh	Avoid injecting into the lower eyelid	0.2
*O2*	Lower lateral orbital	Supraperiosteal^b^	Cannula		Avoid injecting into the lower eyelid	0.2
*O3*	Upper lateral orbital	Supraperiosteal^b^	Cannula		Avoid injecting into the upper eyelid	0.1
Eyebrow (*E*)^h^						
*E1*	Eyebrow tail	ROOF	Cannula			0.2
*E2*	Eyebrow center	ROOF	Cannula		Inject lateral to the supraorbital foramen	0.2
*E3*	Eyebrow head	ROOF	Cannula		Inject lateral to the supratrochlear foramen	0.1
Tear trough (*Tt*)^i^						
*Tt1*	Central infraorbital	Supraperiosteal^b^	Cannula		Be wary of the infraorbital artery branches^i^	0.2
*Tt2*	Lateral infraorbital	Supraperiosteal^b^	Cannula			0.2
*Tt3*	Medial infraorbital	Supraperiosteal^b^	Cannula		Be wary of the angular artery and vein^i^	0.1
Glabella *(G)*^i^						
*G1*	Lateral glabella	Supraperiosteal^b^	Cannula		Be wary of the neurovasculature in the glabellar region, in particular the supratrochlear arteries	0.1
*G2*	Central glabella	Supraperiosteal^b^	Cannula			0.3 (only midline)
*Perioral*						
Nasolabial fold (*NL*)						
Bone deficiency^j^						
*NL1*	Upper nasolabial fold	Supraperiosteal^b^	Needle^j^		Be wary of the facial artery and the branches to the nasal flare^d,^	0.3
Mild/moderate^k^						
*NL1*	Upper nasolabial fold	Subcutaneous	Cannula		Be wary of the facial artery and the branches to the nasal flare	0.3
*NL2*	Central nasolabial fold	Subcutaneous	Cannula		Be wary of the facial artery	0.2
Severe^k^						
*NL1*	Upper nasolabial fold	Subcutaneous	Cannula		Be wary of the facial artery and the branches to the nasal flare	0.5
*NL2*	Central nasolabial fold	Subcutaneous	Cannula		Be wary of the facial artery	0.3
*NL3*	Lower nasolabial fold	Subcutaneous	Cannula		Be wary of the facial artery	0.2
Marionette line (*M*)						
*M1*	Upper marionette line	Subdermal	Needle			0.2
*M2*	Central marionette line	Subdermal	Needle			0.2
*M3*	Lower marionette line	Subdermal	Needle			0.1
Lip (*Lp*)						
*Lp1*	Vermilion body					
*Lp*^*1*^	Upper lip	Submucosa	Cannula			0.2
*Lp*_*1*_	Lower lip	Submucosa	Cannula			0.2
*Lp2*	Cupid’s bow	Mucosa	Needle			0.05
*Lp3*	Lip border					
*Lp*^*3*^	Upper lip	Mucosa	Needle			0.15
*Lp*_*3*_	Lower lip	Mucosa	Needle			0.15
*Lp4*	Medial tubercle	Mucosa	Needle		Be wary of the superior labial artery	0.1 (only midline)
*Lp5*	Lateral tubercles	Mucosa	Needle		Be wary of the inferior labial artery	0.05
*Lp6*	Oral commissure	Mucosa	Needle			0.1
*Lp7*	Philtrum column	Subdermal	Needle			0.05
*Lp8*	Perioral lines					
*Lp*^*8*^	Upper perioral lines	Subcutaneous	Cannula	^l^		0.25
*Lp*_*8*_	Lower perioral lines	Subcutaneous	Cannula	^l^		0.25
*Other*						
Nose (*N*)^h^						
*N1*	Anterior nasal spine (nasolabial angle)	Supraperiosteal^b^	Needle^d^			0.3 (only midline)
		Supraperiosteal^b^	Cannula^m^			0.3 (only midline)
*N2*	Columella (anterior septum)	Cartilage^b^	Needle			0.2 (only midline)
		Cartilage^b^	Cannula^m^			
*N3*	Frontonasal angle	Supraperiosteal^b^	Needle^d^			0.3 (only midline)
		Supraperiosteal^b^	Cannula^m^			
*N4*	Bony dorsum	Supraperiosteal^b^	Needle^d^			0.2 (only midline)
		Supraperiosteal^b^	Cannula^m^			
*N5*	Cartilaginous dorsum	Cartilage^b^	Needle^d^			0.2 (only midline)
		Cartilage^b^	Cannula^m^			

The volume shown in the Active Number column is the recommended volume for injection in one side of the face

ROOF, retro-orbicularis oculi fat; SMAS, superficial muscular aponeurotic system; SOOF, suborbicularis oculi fat; TML, top-model look

^a^Recommended volumes were determined based on the author’s clinical experience with Juvéderm injectables with Vycross technology including Voluma with Lidocaine (Juvéderm Voluma XC), Volift with Lidocaine (Juvéderm Volift XC), and Volbella with Lidocaine (Juvéderm Volbella XC; all, Allergan plc, Dublin, Ireland)

^b^Do not inject into the cartilage or into the bone, but rather *at the level of* the cartilage or *the level of* the bone

^c^*Ck1* is the starting point of every injection with the MD Codes, and its active number is 0.1 + 0.1 + 0.1 mL. These three anchoring points are injected down to the bone to promote SMAS lifting. A single bolus of 0.3 mL in only one site is not advised here as it may bulge and look unnatural

^d^Aspiration is highly recommended when injecting with a needle at the level of the bone

^e^Although the active number for *C1* is 0.5 mL, when treatment of *C1* is combined with *C2*, the active number for *C1* becomes 0.7 mL so that the total volume for *C1 *+* C2 *= 1.0 mL (1 syringe). The same happens when *Ck1* is combined with *T1* or *Ck4*

^f^Mainly used in male patients

^g^This approach is for restoring forehead volume loss, forehead advancement, and forehead reshape

^h^Treatment of the forehead, eyebrow, glabella, and nose areas is very advanced and should only be delivered by highly trained experts with extensive injection experience and knowledge on the management of severe complications

^i^Tear trough and orbital codes are reserved for specialists specifically trained in this technique and those who have a sound knowledge of the anatomy and physiology for this particular area

^j^This treatment approach is designed to correct bone structural deficiencies

^k^The use of cannulas in the nasolabial fold is advisable to correct dynamic nasolabial folds. The use of needles at the deep dermal level may be used for the correction of fine lines

^l^*Lp8* may also be treated using micro-aliquot injections with needles at the subdermal level

^m^Small and low noses may be better addressed with cannulas

= bolus, static injection of injectable (0.3 mL);  = linear injection (anterograde or retrograde; 0.5 mL);  = fanning, defined as multiple linear injections via a single entry site creating a fan-like pattern with cannulas (0.5 mL);  = micro-aliquot injections of very small droplets of injectable (0.01–0.05 mL per point);  = aliquot injections, defined as static injections of a small amount of injectable (0.1–0.2 mL)

Shapes associated with the codes for HA fillers indicate injection delivery (e.g., bolus or linear injection; Table 3). For each MD Code, there is also an associated target injection depth (e.g., subcutaneous or supraperiosteal), a tool for product delivery (needle or cannula), and a minimal volume of product to inject (active number). The use of needles is preferable for precise bolus injections at the level of the bone and/or when precision and definition is required to treat fine lines in the subdermal plane (e.g., for lip lines). The use of cannulas is preferred for subcutaneous and fat pad injections and when the proximity of vessel bundles is a concern.

## MD Codes Equations

A set of MD Codes that prescribes the treatment of a specific facial deficiency is grouped to form an equation (Table 4). For example, the equation to treat the tear trough area is *Tt1 *+* Tt2 *+* Tt3*, where each code denotes the facial unit (Tt) and subunit (1, 2, or 3). However, direct treatment of the tear trough area, or any deficiency, in isolation is not ideal and may lead to patient dissatisfaction and adverse events. Rather, the treatment of the tear trough area should be planned and carried out in the context of the unfavorable facial messages to which tear troughs contribute, including equations for each deficiency. Saggy cheeks, sunken temples, and eye bags may contribute to a tired appearance and, therefore, the treatment of a tired look may require MD Codes equations for each of those deficiencies.

**Table 4:** Checklist of standard equations for treating facial deficiencies with the MD codes

Structural component addressed	Facial deficiency	Equation
Foundation, midface	Saggy cheeks/cheek-volume loss	*Ck1 *+* Ck2 *+* Ck3 *+* Ck4 *+* Ck5*
Contour, upper face	Sunken temples	*T1 *+* T2*
Contour, lower face	Small/recessed chin	*C1 *+* C2 *+* C3 *+* C4 *+* C5*^a^+* C6*
	Jowls/double chin	*Jw1 *+* Jw2 *+* Jw3 *+* Jw4 *+* Jw5*
Refinement, periorbital	Volume loss in forehead	*F1 *+* F2 *+* F3*
	Low brows	*E1 *+* E2 *+* E3*
	Volume loss in lateral orbit	*O1 *+* O2 *+* O3*
	Tear trough	*Tt1 *+* Tt2 *+* Tt3*
Refinement, perioral	Deep nasolabial folds	*NL1 *+* NL2 *+* NL3*
	Lack of lip structure/lip volume loss	*Lp1 *+* Lp2 *+* Lp3 *+* Lp4 *+* Lp5 *+* Lp7 *+* Lp8*
	Downturn of oral commissures	*Lp6*
	Marionette lines	*M1 *+* M2 *+* M3*
Refinement, nose	Nose reshape	*N1 *+* N2 *+* N3 *+* N4 *+* N5*

Clinicians should tailor each equation to the needs of the patient (see Table 1)

*C* chin, *Ck* cheek, *E* eyebrow, *F* forehead, *Jw* jowl, *Lp* lip, *M* marionette, *N* nose, *NL* nasolabial, *O* orbit, *T* temple, *Tt* tear trough

^a^For male patients

The order in which the series of MD codes equations are addressed may significantly affect treatment success. In the author’s experience, patient satisfaction is improved when planning and implementation of treatment is conducted in a specific sequence, according to a principle of foundation, contour, and refinement. This approach has been described using the analogy of the construction of a house [19]; laying a foundation is always the first step, followed by contouring, or constructing the framing, floors, and walls. Refinements, such as interior decor, are added last. When treating the face, the foundation is laid by creating structure and reducing sagginess in the midface. Treatment of the cheek area should always begin by addressing the lateral lifting vectors, represented by *Ck1* and *Ck4*. The contour step is divided into the upper face (treating the temples) and the lower face (chin and jawline). The final step is refinement, which involves treating tear troughs and lateral canthal lines (or crow’s feet lines) in the periorbital area, and deep nasolabial folds, the lips, and marionette lines in the perioral area. Thus, treating a tired look should be addressed by first providing foundation to the midface (e.g., saggy cheeks: *Ck1 *+* Ck2 *+* Ck3 *+* Ck4*), then contouring the upper face (e.g., sunken temples: *T1 *+* T2*), and, finally, refining the periorbital area (e.g., tear troughs: *Tt1 *+* Tt2 *+* Tt3*).

### Algorithms for Selecting MD Codes

**Fig. 2:**
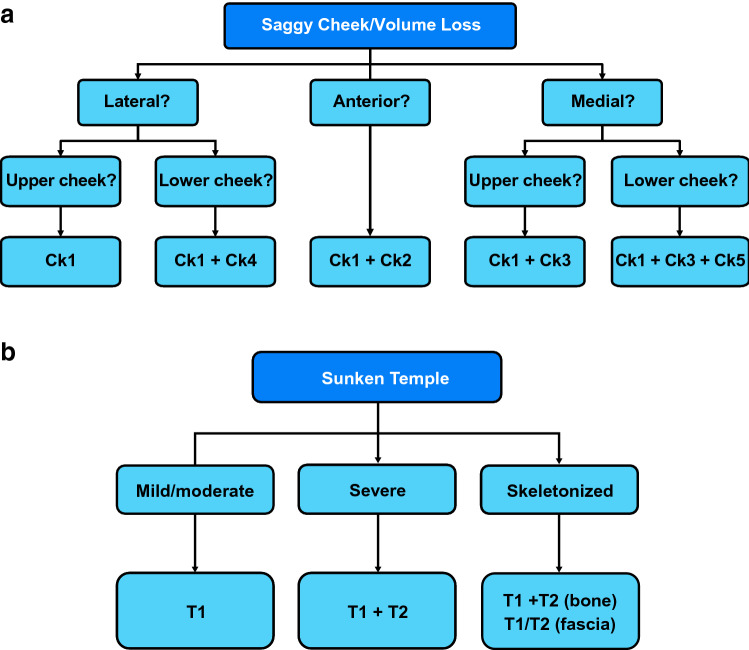

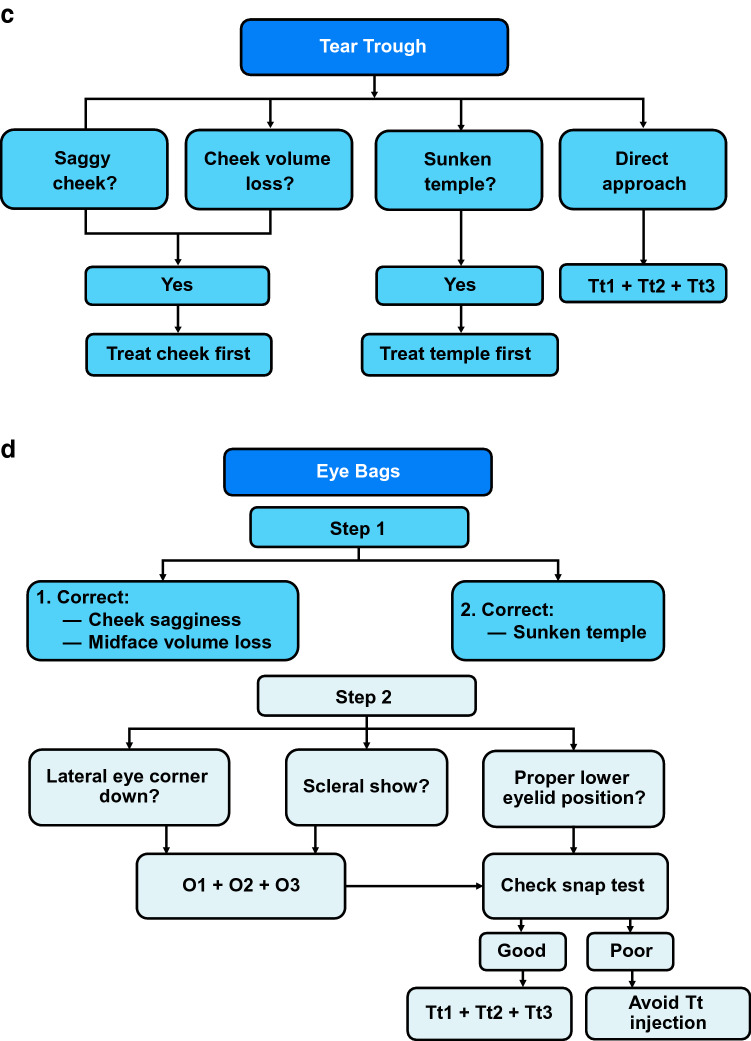
Algorithm for deciding which MD Codes to use to provide midface foundation by (**a**) treating saggy cheeks, to contour the upper face by (**b**) treating sunken temples, and to provide periorbital refinement by treating (**c**) the tear troughs and (**d**) the eye bags. *Ck1,* zygomatic arch; *Ck2,* zygomatic eminence; *Ck3*, anteromedial cheek–midcheek; *Ck4,* lateral lower cheek/parotid area; *Ck5,* submalar area; *T1*, anterior temple; *T2*, posterior temple; *Tt1*, central infraorbital; *Tt2*, lateral infraorbital; *Tt3,* medial infraorbital. Reprinted with permission from Maurício de Maio, MD, PhD

To handle the enormous variability among faces, the MD Codes algorithms were developed to guide selection of the appropriate MD Codes for each individual. Not every patient will need all of the codes within each equation. For example, the algorithm for saggy cheeks (Fig. 2a) guides the selection of MD Codes based on the presence or absence of volume loss. When comparing a daughter, a mother, and a grandmother, the daughter may have fullness in her cheek area and present with only a mild degree of sagginess. Thus, according to the saggy cheeks algorithm, she would benefit from treatment of only *Ck1*. The mother may present with sagginess and volume loss concentrated only in the medial aspect. She may benefit from treatment of *Ck1*, *Ck3,* and *Ck*4. The grandmother, due to a greater severity of volume loss and sagginess, may be eligible for treatment of all five cheek anatomical areas (*Ck1* + *Ck2* + *Ck3* + *Ck4* + *Ck5*).

Someone with a tired look may also present with sunken temples. The algorithm for sunken temples (Fig. 2b) guides clinicians on the selection of MD Codes based on the severity of volume loss in the temples. In the case of the daughter, the mother, and the grandmother, the daughter may not need any temple treatment as she may have no deficiency there. The mother may present with mild to moderate volume deficit in the temples and may benefit only from treatment of *T1*. The grandmother, with a more severe temple deficiency, may benefit from treatment of *T1* and *T2*.

The algorithms for treatment of the tear troughs and the eye bags are shown in Fig. 2c and d, respectively. The vast majority of people presenting with distracting tear troughs and eye bags also have saggy cheeks and/or volume loss that would first require treatment of the cheeks (foundation) and the temples (contouring). Only a young patient who presents with no volume loss or sagginess may benefit from direct treatment of the tear troughs (*Tt1 *+* Tt2 *+* Tt3*), but this rarely occurs in clinical practice.

Combining the MD Codes identified using the three algorithms to treat a tired look may result in, for example, a total of seven MD Codes for the mother ([*Ck1 *+* Ck3* +* Ck4] *+* [T1] *+* [Tt1 *+* Tt2 *+* Tt3*]), and 10 codes for the grandmother ([*Ck1* + *Ck2 *+* Ck3 *+* Ck4 *+* Ck5] *+* [T1 *+* T2] *+* [Tt1 *+* Tt2 *+* Tt3*]). Thus, the difference between the treatment plans for the mother and the grandmother is in the number of codes, based on degree of severity; the grandmother does not necessarily receive more volume per code, which could lead to unnatural results and adverse events. Once the MD Codes are selected for the individual patient, a tired look is addressed step by step, first providing foundation in the midface, then contouring at the temples, and, finally, directly addressing the tear troughs or eye bags as refinement. Additional MD Codes algorithms are provided in Figure S1 in the Supplemental Materials. Clinicians must explain to patients who focus only on the refinement step that the foundation and contour should be addressed first, as represented in these algorithms.

**Fig. 3:**
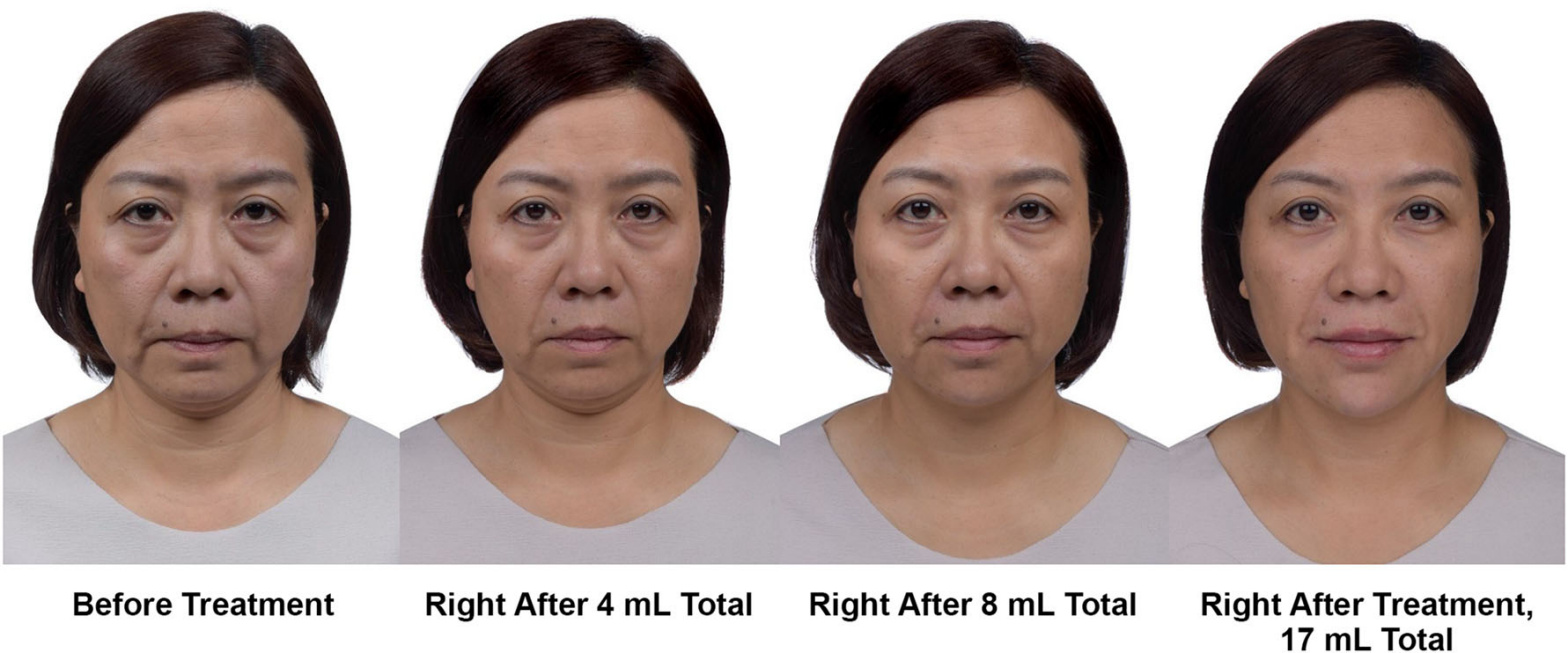
Improvement in emotional attributes of the face, with increasing volume over successive treatment steps using the MD Codes. This 53-year-old Asian woman presented with facial signs of sagginess in the midface and lower face, a tired look, and a sad look (left image). She is shown before treatment and immediately after receiving 4 mL total, 8 mL total, and the full 17 mL treatment, within a single session. The patient received a total of 17 syringes of Juvéderm products (Allergan plc), administered as 13 mL of Voluma, 3 mL of Volift, and 1 mL of Volbella. Reprinted with permission from Maurício de Maio, MD, PhD

Over the foundation, contour, and refinement steps, the MD Codes approach can result in the progressive removal of the unfavorable attributes of tiredness, sadness, and sagginess, and may enhance the positive attributes of looking younger and more feminine. Figure 3 illustrates the change in appearance that may be observed as additional MD Codes are used to add volume in successive treatment steps. Notice the improvement in the patient’s cheek immediately after providing midface foundation (*Ck1 *+* Ck2 *+* Ck3 *+* Ck4*) and contouring of the upper face (*T1*) with a total 4 mL of HA filler. The patient’s jawline was improved after contouring of the lower face with an additional 4 mL of HA filler (*C1 *+* C2 *+* Jw4 *+ *Jw5*). Improvement in her double chin was also achieved by addressing the cheek first, then contouring the chin and anterior jawline. Foundation and contour were reinforced with additional codes (*Ck3 *+* C6*), refinement of the periorbital area improved the tear troughs (*Tt1 *+* Tt2 *+* Tt3*), and perioral refinement addressed the lips and nasolabial folds (*Lp*_*1*_ + *Lp2* + *Lp*^*3*^ + *Lp*_*3*_ +* Lp5 *+* Lp6 *+* NL1*) using a final 9 mL of filler. The image on the right in Fig. 3 shows the patient immediately after the total injection volume of 17 mL. Increasing the number of MD Codes over these multiple treatment steps yielded more impactful results. This patient received all treatment steps in a single session; however, the author suggests planning treatment such that 4 mL is injected per session. To achieve the best possible results, more volume may be provided by using additional codes in successive sessions to reinforce facial restructuring in the midface, cheek, and jawline.

### Volume Planning: The MD Codes Active Numbers

One of the biggest challenges clinicians may encounter during treatment planning is estimating the total volume of product required for treatment. Often a clinician may discover mid-treatment that the planned number of syringes is not adequate. When fillers were first introduced to the market, it was common for clinicians to treat only nasolabial folds or to distribute the product of one syringe (1 mL) among many sites, leading to a lack of visible results and patient dissatisfaction. Clinical judgment should be used to determine the appropriate total volume based on individual patient needs; however, a recommended injection volume, or active number, is provided with each MD Code to simplify the estimation of the total volume. The active numbers are minimum volumes needed to achieve visible and reproducible results (Table 3); actual injection volumes should be determined by the clinician for each patient.

The analogy of building a house is again useful for understanding the importance of volume planning. The amount of material to be used in each stage of construction is critical and must be carefully calculated in advance, in order to create a financial plan and ensure that the structure will be sound. Similarly, clinicians must know beforehand how many syringes they will need for each treatment step, to be able to communicate accurate cost information to the patient and to achieve optimal and long-lasting results.

Volume recommendations provided here (and to be presented in greater detail in a future publication) were established progressively by the author based on clinical experience with more than 10,000 syringes over a 4-year period. Clinicians may follow a simple rule: bolus injections should not exceed a total volume of 0.3 mL into the same compartment, to avoid disturbing muscle movement. There are three exceptions to this rule: when injecting the temple (*T1* + *T2*) and the mandible angle (*Jw1*), the active numbers are 0.5 mL. When fanning in a specific area with a cannula, no less than 0.5 mL should be used.

To estimate the product volume needed during treatment planning, clinicians should first determine the MD Codes required for their patient, using the algorithms as described above. The active numbers for each code are then summed to estimate the minimum total volume required. For example, treating a tired look may require the following MD Codes:

**Table:** 

*Ck1*	0.1 mL + 0.1 mL + 0.1 mL	= 0.3 mL
*T1*	0.7 mL	= 0.7 mL
*Ck2*	0.2 mL	= 0.2 mL
*Ck3*	0.3 mL	= 0.3 mL
*Tt1 *+* Tt2 *+* Tt3*	0.2 mL + 0.2 mL + 0.1 mL	= 0.5 mL
	Total volume per one side of the face	= 2.0 mL
	Total volume for both sides of the face (2 × 2.0 mL)	= 4.0 mL

Note that when both *Ck1* and *T1* are injected during the same session, the active number for *T1* is increased from 0.5 to 0.7 mL to allow for use of an entire 1-mL syringe. For this specific treatment, the clinician will need a total of four syringes, two for each side of the face. All patients whose treatment plans include the same MD Codes will start at the same estimated minimum total volume. In this way, the MD Codes approach reduces variability in outcomes by focusing on facial attributes rather than on the differences in age, gender, and ethnicity in the faces that harbor them.

## Safety

The MD Codes were designed to provide guidelines for effective placement of HA fillers; future large-scale studies with MD Codes are needed to verify the rate of adverse events in comparison with injectable filler treatment in general. Most commonly reported complications with injectable filler treatment include injection site reactions, such as swelling, bruising, redness, and pain [20–23]. Reported rates of complications range widely in a recent systematic review of 22 studies of HA fillers; rates of swelling, bruising, and lumps or bumps ranged from less than 10% to more than 90% across trials [23]. Few studies reported the severity of adverse events, but in those that did, most events were mild (71–88%) or moderate (11–16%) [23]. More serious complications are rare but do occur. Delayed inflammatory reactions, examined in an analysis of 35 studies, were found to occur at a rate of 1.1% per year based on patient month at risk [24]. A review of severe complications in the literature reported 22 articles describing necrosis or impending necrosis in patients treated with HA fillers, most commonly following injections in the nose, nasolabial folds, or glabella [25]. Vision loss or blindness may also occur on rare occasions, likely due to blockade of ophthalmic circulation via the ophthalmic artery by filler emboli; 44 cases of partial or complete blindness after HA filler injection were described in a review of vision loss associated with HA filler treatment [26]. Vision loss most commonly resulted from injections in the area of the nose but followed treatment at a range of upper and midface sites.

In the author’s practice, no serious complications, such as arterial embolic accident or necrosis, have occurred. Among 387 treated patients (in which the average number of syringes injected was 14), the most frequent adverse event was mild or moderate localized edema (28 patients), occurring within the first 2 months after the procedure in the majority of those patients reporting this event; delayed edema occurred in few cases. Most patients received a corticosteroid and an antihistamine prior to treatment, which may have contributed to low rates of injection site reactions. Edema generally subsided within 2 h, without additional treatment. When needed, antihistamines were used as a first medication therapy and, if unresolved, prednisolone (40 mg/day for 5 days) was prescribed. Delayed edema was usually associated with a trigger factor, such as sinusitis, a cold, or fatigue; patients with multiple allergies were also at higher risk. Rarely, hyaluronidase was needed to dissolve the product.

Although the MD Codes denote alert areas, they are designed only to remind clinicians of areas where extreme caution is required and do not eliminate the risk of complications when those areas are addressed. Clinicians must consider the risk–benefit ratio in providing facial aesthetic treatment, and only highly trained experts who have a deep understanding of the anatomy and physiology of an alert area should attempt treatment of a hazardous area.

## Case Presentations

### Case Study 1

**Fig. 4:**
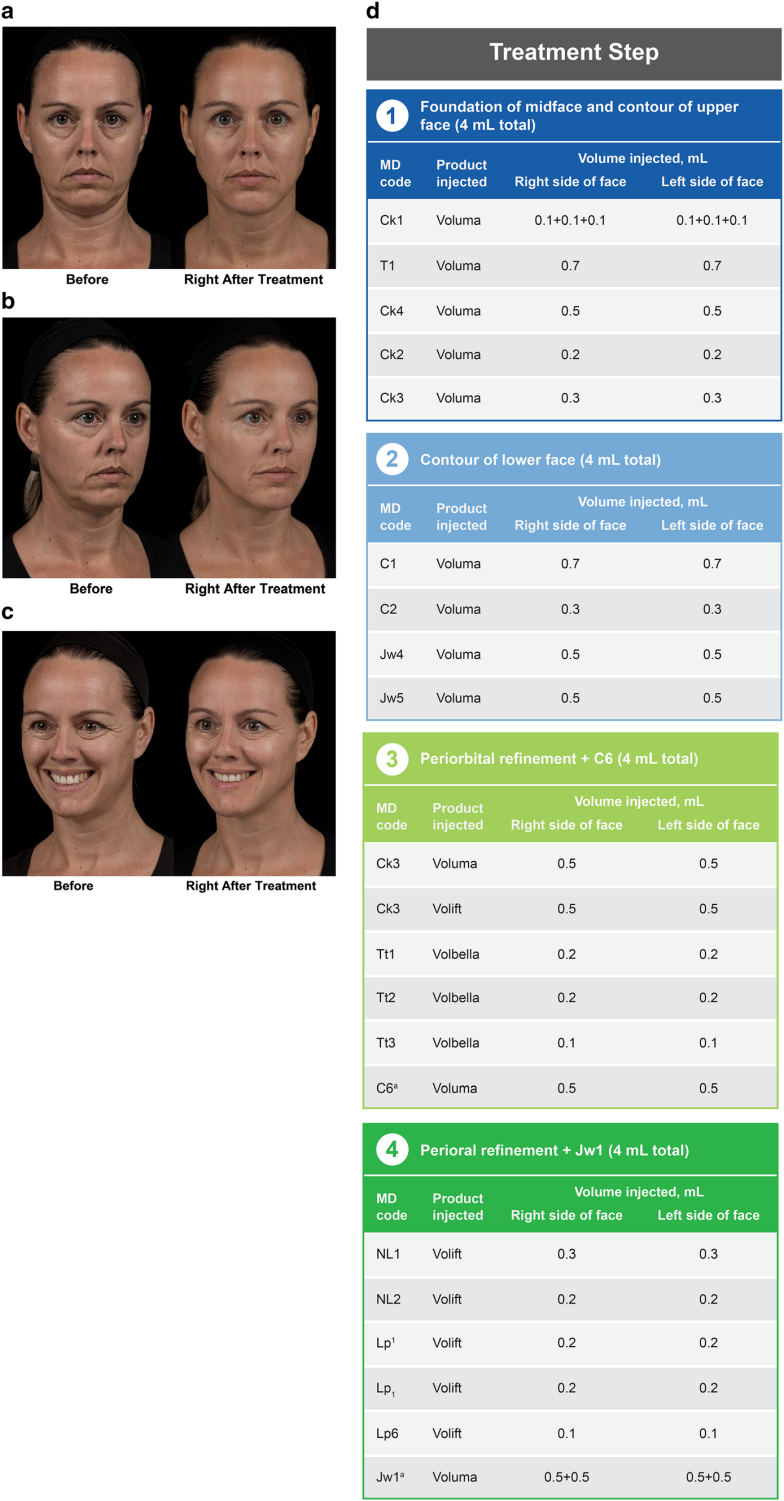
*Case 1:* A 43-year-old woman with a saggy appearance in the midface and lower face, tiredness around the eyes, and sadness in the periorbital and perioral areas. Images show the patient before treatment and immediately after the last treatment step from (**a**) the frontal view and from the oblique view, with (**b**) neutral and (**c**) animated facial expressions. The MD Codes, volumes, and products injected are summarized in panel **d**. The patient received Juvéderm products administered as 12 mL of Voluma, 3 mL of Volift, and 1 mL of Volbella, with a total of 16 syringes injected. ^a^The patient needed additional treatment of *C6* and *Jw1* during the third and fourth steps to provide additional contour to the lower face. Reprinted with permission from Maurício de Maio, MD, PhD

A 43-year-old Caucasian woman (Fig. 4) presented with signs of sagginess in the midface and lower face, looking tired around the eyes and looking sad around the eyes and mouth. The first step of treatment provided foundation in the midface by addressing the cheek codes. The algorithm for saggy cheeks was used to select the MD Codes for injection and, as mentioned previously, always starts with the lateral lifting cheek vectors (*Ck1 *+* Ck4*) and proceeds to the anterior (*Ck2*) and medial (*Ck3*) cheek. Contour was then provided to the upper face by addressing the volume deficit in the temple (*T1*), which indirectly improved the appearance of her eye bags. Next, the lower face was contoured by injecting *C1* to improve the labiomental angle, *C2* to increase the vertical height of the chin, and *Jw4* and *Jw5* to advance the mandible and to improve the prejowl area. Midface foundation was reinforced by injecting *Ck3* with two different approaches, first the deep malar fat pad, then the suborbicularis oculi fat (SOOF). The lateral aspects of the chin and prejowl areas were also addressed by injecting *C6*. Finally, periorbital refinement was provided in the tear trough area (*Tt1 *+* Tt2 *+* Tt3*). Refinement in the perioral area was provided by addressing the nasolabial folds (*NL1 *+* NL2*) and lip volume (*Lp*^*1*^ +* Lp*_*1*_ +* Lp*^*6*^). Lastly, *Jw1* was injected to reshape the mandible angle.

Images taken immediately after treatment illustrate improvements in the patient’s midface, jawline, periorbital, and perioral areas (Fig. 4a–c). The reshape of the jawline apparent in the oblique view (Fig. 4b) was accomplished by contouring with injections in *C1, C2, Jw4, Jw5, C6,* and *Jw1*. In the oblique view, improvement of the eye bags was observed after injection of *Ck1, T1, Ck2, Ck3* and *Tt1, Tt2, Tt3*. The periorbital lines improved after addressing periorbital deflation. In the photographs taken immediately after treatment, you can see that using the MD Codes algorithm to select codes and focusing treatment on foundation, contour, and refinement resulted in the elimination of the unfavorable tired, saggy, and sad attributes of the face and enhancement of the positive attributes of looking younger, more feminine, and attractive. This patient (and the following 2 cases) received all injections in a single session, for educational purposes. However, in clinical practice, this treatment could be provided in multiple sessions, with four syringes of HA filler injected during each session.

### Case Study 2

**Fig. 5:**
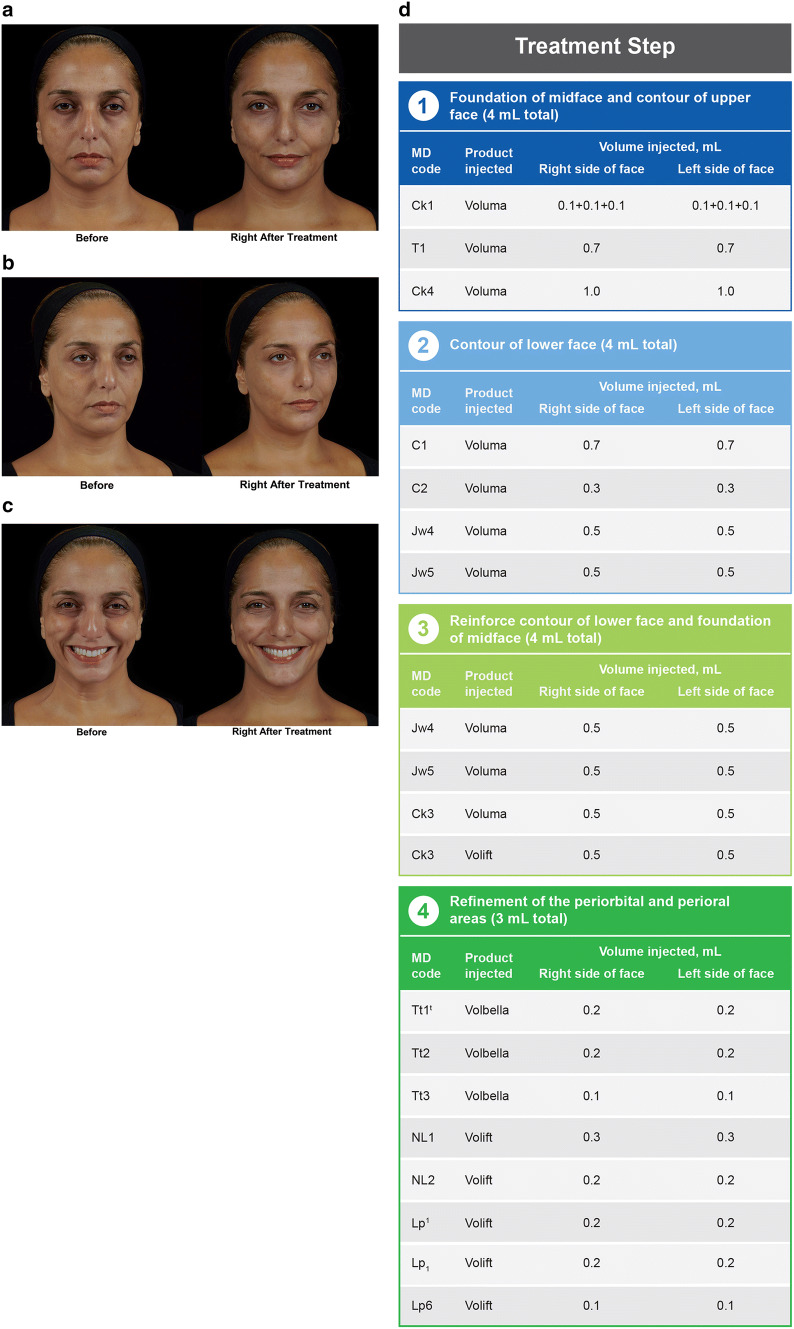
*Case 2:* A 44-year-old women with facial signs of sagginess, tiredness, and sadness represented by the presence of saggy cheeks, eye bags, and marionette lines. Images show the patient before treatment and immediately after the last treatment step from (**a**) the frontal view and from the oblique view, with (**b**) neutral and (**c**) animated facial expressions. The MD Codes, volumes, and products injected are summarized in panel (**d**). The patient received 11 mL of Voluma, 3 mL of Volift, and 1 mL of Volbella, with a total of 15 syringes injected. Reprinted with permission from Maurício de Maio, MD, PhD

A 44-year-old woman (Fig. 5) presented with facial signs of sagginess, tiredness, and sadness, represented by the presence of saggy cheeks, eye bags, and marionette lines. Before treatment, indentation was apparent on the anterior right cheek when she smiled (Fig. 5c). The first treatment step focused on providing foundation in the midface (*Ck1 *+* Ck4*) and contouring the upper face (*T1*). The second step addressed the lower face by treating the chin (*C1 *+* C2*) and anterior jawline (*Jw4 *+* Jw5*). In step 3, due to the degree of severity and to further improve the jawline, *Jw4 * and *Jw5* were reinjected. In the same step, *Ck3* was injected to compensate for the deep malar fat pad and then the SOOF. The final step included periorbital and perioral refinement through injections in the tear trough area (*Tt1 *+* Tt2 *+* Tt3*), the nasolabial folds (*NL1 *+* NL2*), and lip (*Lp*^*1*^ +* Lp*_*1*_ +* Lp6*). Images taken immediately after the last treatment step illustrate the changes in facial shape, including increased volume in the cheeks, and the more triangular appearance of the chin (Fig. 5a). After the lip treatment, an upturn of the oral commissures was observed. In the oblique view, the improvement of saggy cheeks, eye bags, and the jawline resulted in a less tired, less saggy, and less sad appearance than before treatment (Fig. 5b). Injection of *CK1* and *Ck4* improved cheek sagginess and reduced the indentation of the woman’s right cheek, making her smile appear more attractive and natural (Fig. 5c).

### Case Study 3

**Fig. 6:**
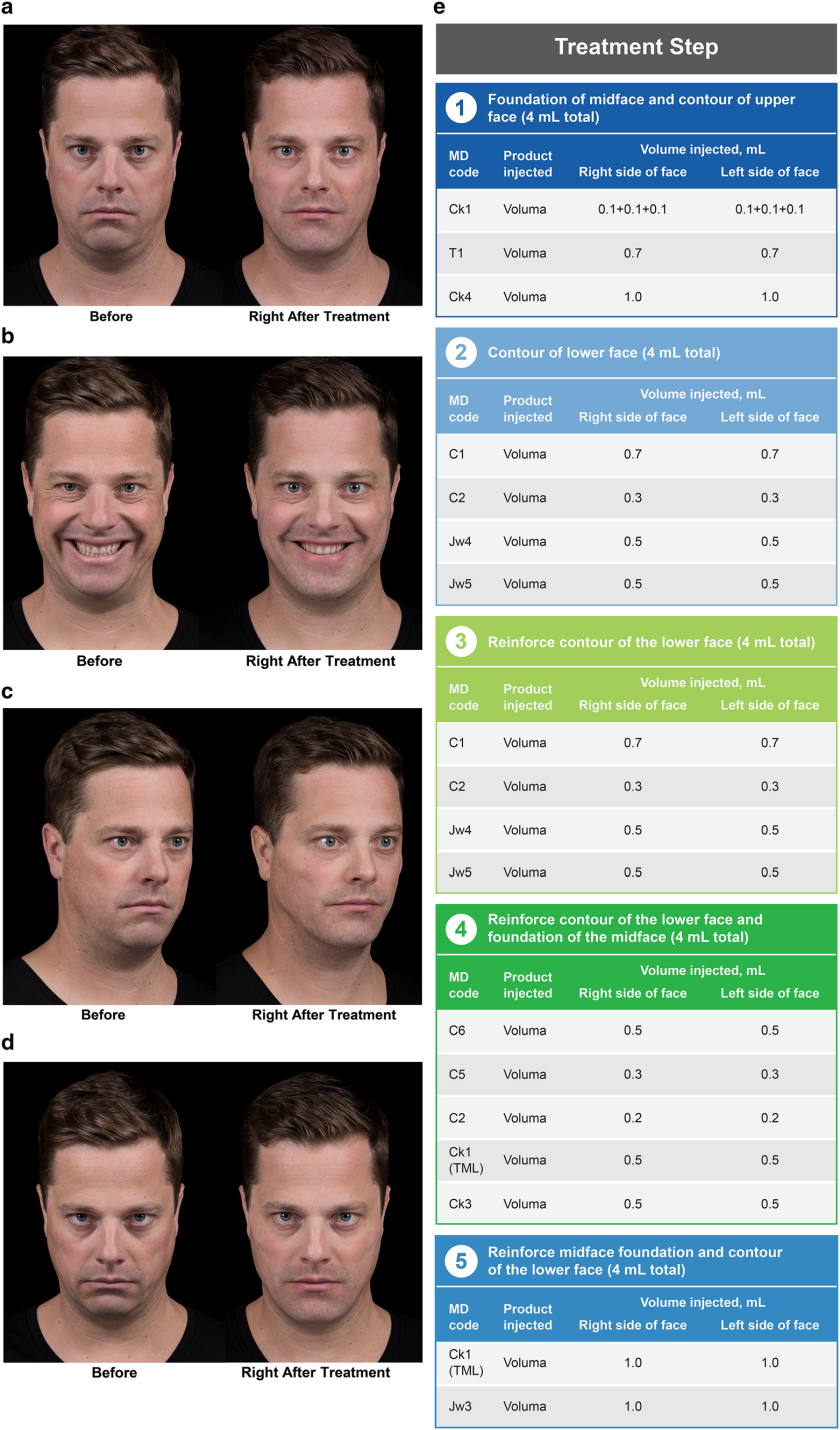
*Case 3*: A 42-year-old man with saggy cheeks, a poorly defined jawline, and a double chin. Before and after images are shown from the frontal view with (**a**) neutral and (**b**) smiling expression, from (**c**) the oblique view, and from (**d**) the frontal view with the head tilted downward. The MD Codes, volumes, and products injected are summarized in panel **e**. The patient received a total of 20.0 mL of Juvéderm Voluma. Reprinted with permission from Maurício de Maio, MD, PhD

A 42-year-old Caucasian man (Fig. 6) presented with a narrow face, saggy cheeks, a poorly defined jawline, and a double chin. The first treatment step focused on the same lateral lifting vectors as in case 2 (*Ck1 + T1* + *Ck4*) to build midface foundation and to contour the upper face. Step 2 focused on improving chin projection by treating the chin (*C1 *+* C2*) and jawline (*Jw4 *+* Jw5*) in the same manner that treatment was delivered for cases 1 and 2. For this patient, repeat treatment was required in a subsequent session due to the degree of severity. The lateral chin area and prejowl sulcus (*C6*), the lateral lower chin (*C5*), and the chin apex (*C2*) were then addressed. In addition, *Ck1* was injected for the top-model look (TML; *Ck1*), which refers to the injection of *Ck1* with a cannula to create a linear appearance of the zygomatic arch. *Ck3* was injected to address the deep malar fat pad. Injection of *Ck1* (TML) was repeated in a later session to further reinforce the zygomatic width, and *Jw3* was injected to sharpen the jawline. Immediately after treatment, changes in facial shape could be observed, with improvements in the bitemporal, bizygomatic, and bigonial widths due to the injections of *T1, Ck1,* and *Jw1,* respectively (Fig. 6a). Indirect improvements to the tear trough area could also be observed following the injection of *Ck1, T1,* and *Ck3*. The chin presented with a more square shape, and there was less indentation of the labiomental sulcus and the prejowl area. Note that *C1, C2, C5,* and *C6* were injected, as well as *Jw4* and *Jw5,* to improve chin shape and address the double chin. When the patient smiles, a reduction in his double chin and smile lines can be seen (Fig. 6b). His smile looks more confident due to improvements in the lateral structure of the face (*Ck1 *+* T1 *+* Ck4*) and support of the deep malar fat pads (*Ck3*). Injection of *C1* also lifted the lower lip and lessened showing of his lower teeth when smiling. The oblique view (Fig. 6c) shows improved definition of the zygomatic arch resulting from *Ck1* (TML), a better jaw angle due to treatment of *Jw1,* a more square chin shape due to treatment of *C1, C2, C5,* and *C6,* and a more defined jawline due to treatment of *Jw3*. When the patient looked down, the improvement of facial shape and increased stability in the lower lip helped to create a more confident appearance (Fig. 6d).

## Discussion

The approach to treatment with facial fillers has historically focused on improving the appearance of lines and folds found distracting by the patient. The MD Codes approach was not designed to treat individual deficiencies within the patient’s face, but to focus instead on the emotional attributes that the entire face presents. By addressing the messages of the face and sequencing treatment using the principle of foundation, contour, and refinement, the clinician can deliver improvement beyond the elimination of isolated deficiencies to meet patients’ true expectations for a more global transformation.

The MD Codes specify the injection location, depth, volume, and device, allowing clinicians to reduce variability in those technical aspects that can be precisely controlled. However, there will always be variability in patient outcomes because each person’s face presents a different starting point. Using the same MD Codes will not cause all patients to look the same after treatment, as illustrated in the presented case studies. Two of these patients received treatment using the same MD Codes, and each achieved improvement in the unfavorable messages their faces conveyed. Further, results will likely vary with the level of a clinicians’ expertise. The clear and standardized instruction of the MD Codes on how HA fillers should be injected may help novice clinicians reduce variability in their results to achieve successful outcomes and patient satisfaction, and may optimize success rates in experienced clinicians.

Patient education during the treatment planning stage, establishing with each patient the larger goals of treatment and the process by which they can be achieved, is also critical for successful outcomes. Patients tend to focus on the refinement step. The clinician can educate the patient on the importance of providing foundation and contour first using the house building analogy. With the MD Codes and their active numbers, clinicians can help patients to understand the components of their treatment plan and to have well-informed expectations for treatment outcomes. The active numbers allow clinicians to estimate in advance the adequate volume of product required to treat patients according to their emotional attributes. They provide a tool for financial planning and may help to ensure that the entire contents of a syringe will be used, minimizing waste and overall costs and reducing the risk of infection.

The MD Codes may also help to eliminate language barriers in the development of best practices in aesthetic treatments. They are easy to learn, do not require the ability to speak or read a specific language, and reduce the amount of text required for planning and implementing treatment. The MD Codes can thus facilitate clear communication between clinicians and help newer clinicians to achieve quality, reproducible results. The components of the MD Codes are summarized for ease of use in flash cards developed to communicate the technique (Figure S2). In the future, the MD Codes may prove useful for reducing variability across clinicians within clinical studies to more objectively evaluate new products and new areas of treatment.

Although the ideas within this paper represent the opinions of the author, the MD Codes approach was developed based on extensive experience treating patients worldwide; clinicians around the world have found the MD Codes and algorithms to be effective and are using them in clinical practice and educational settings [27, 28]. The MD Codes system has not been independently supported by clinical trial data. Injection volumes suggested by the active numbers, based on the author’s clinical experience with a worldwide patient population, are intended as a starting point for the treatment plan, to be tailored to each patient. The author has not used patient-reported outcome scales to precisely measure patient satisfaction with treatment in clinical practice, and there are, as yet, no objective measures to support this approach for optimizing outcomes. No safety data for treatment using the MD Codes are available. Common adverse events associated with HA filler treatment, including injection site reactions, should be expected [29–31]. Real-world experience using the MD Codes across multiple clinicians will be invaluable in assessing the practical utility of this clinical tool, but comparative studies should be developed to assess the relative safety and efficacy of treatment administered using the MD Codes system.

## Conclusions

There is a need to focus the approach to injectable HA filler treatments worldwide on delivering more natural-looking results and optimal patient satisfaction. The MD Codes are a set of shapes, colors, and numbers that provide a universal language for clear and objective guidelines. They are designed to reduce treatment variability, increase clinician success rates, and facilitate financial planning. While years of experience with HA filler treatments and thorough knowledge of facial anatomy may have the greatest impact on treatment outcomes, the MD Codes may optimize the performance of the novice clinician and enhance the success of clinicians with more experience. To improve patient satisfaction, treatment must go beyond the elimination of isolated lines and folds to define success as a reduction in negative attributes and enhancement of positive attributes. By sequencing treatment with MD Codes according to the principle of foundation first, contour second, and refinement last, clinicians may deliver next-level HA filler treatment focused on the emotional messages of the face.

## Electronic supplementary material

Below is the link to the electronic supplementary material.

Supplementary material 1 (DOCX 26 kb)

Supplementary material 2 (EPS 668 kb)

Supplementary material 3 (EPS 534 kb)

Supplementary material 4 (EPS 576 kb)

Supplementary material 5 (EPS 744 kb)

Supplementary material 6 (EPS 735 kb)

Supplementary material 7 (EPS 633 kb)

Supplementary material 8 (TIFF 666 kb)

Supplementary material 9 (TIFF 2858 kb)
