# Supplementary material for: MD Codes™: A Methodological Approach to Facial Aesthetic Treatment with Injectable Hyaluronic Acid Fillers
Source: Aesthetic Plast Surg. 2020 May 22;45(2):690–709. doi: 10.1007/s00266-020-01762-7 (PMC8012343; doi:10.1007/s00266-020-01762-7)
Supplement: Supplementary file 1 — Supplementary material 1 (DOCX 26 kb) [file 266_2020_1762_MOESM1_ESM.docx]

**SUPPLEMENTAL MATERIALS**

**Table S1**. MD Codes Injection Areas and Effects of Injection.

| Anatomical Unit  MD Code | Injection Area | Effect of injection |
| --- | --- | --- |
| **Foundation** | | |
| Cheek *(Ck)* , 5-point cheek reshape | |  |
| *Ck1* | Zygomatic arch | - **A lifting code:** lifts and defines the cheek - Gives support to the eyebrow and lower eyelid, and opens the eye - Improves sagginess of the cheek and jawline - Indirectly improves hollowness in the infraorbital area and tear trough - Indirectly improves prominent nasolabial folds, oral commissures and marionette lines; may aid in creating a slimming and more contoured effect |
| *Ck1 TML* | Zygomatic arch | - Creates structure and definition along the zygomatic arch - May aid in creating a sophisticated look in the upper cheek |
| *Ck2* | Zygomatic eminence | - **A lifting code:** lifts and provides projection to the cheek - Shortens the palpebral-malar sulcus and gives support to the lateral canthal area - Improves eye shape - Indirectly improves nasolabial folds |
| *Ck3* | Anteromedial cheek | - **A volumetric code:** creates volume, projection, and improves the medial lid–cheek junction by advancing the midcheek - Lifts the cheek - Indirectly softens the tear trough and nasolabial fold |
| *Ck4* | Lateral lower cheek/parotid area | - **A lifting code:** lifts the lower cheek and jawline - Addresses the sunken area at the parotid level - May aid in creating a slimming and more contoured effect |
| *Ck5* | Submalar/buccal area | - **A volumetric code:** improves volume loss in the submalar/buccal area - Corrects collapse of the buccal area and improves cheek lines |
| **Contour** |  |  |
| Upper face | | |
| Temple *(T)* , 2-point temple reshape | |  |
| *T1* | Anterior temple | - Reshapes the temple - Reduces the concavity at this level - May indirectly lift the eyebrow |
| *T2* | Posterior temple |  |
| *T1/T2* | Anterior and posterior temple | - Additional reshaping for the temple for those with a skeletonized appearance |
| Lower face |  |  |
| Chin *(C)*, 6-point chin reshape | | |
| *C1* | Labiomental angle | - Opens the labial-mental angle - Reduces the mental crease - Elongates the chin - Reduces the protrusion of the lower lip - Gives support to the oral commissure |
| *C2* | Chin apex | - Improves vertical dimension - Improves chin height - Makes the chin triangular |
| *C3* | Anterior chin | - Improves anterior projection of the chin |
| *C4* | Anterior chin/soft tissue (pogonion) | - Creates the labial-mental sulcus - Rotates the chin upward - Shortens chin height - Enhances *C2* and *C3* - Opposite effect of *C1* |
| *C5* | Lateral lower chin | - Gives lateral support - Widens the chin - Makes the chin square - Ideal for men |
| *C6* | Lateral chin | - Improves lateral chin depression - Reduces the prominence of the prejowl sulcus - Gives support to the marionette line area |
| Jowls *(Jw), 5-point jawline reshape* | |  |
| *Jw1* | Mandible angle | - Defines the mandible angle - Improves mandible projection - Widens the face for masculine-looking results |
| *Jw2* | Pre-auricular area | - Lifts the lateral cheek - Reduces jawline sagginess - Reduces pre-auricular lines |
| *Jw3* | Mandible body | - Improves definition of the jawline |
| *Jw4* | Lower prejowl | - Corrects prejowl sulcus - Lifts the jawline - Improves submental skin excess |
| *Jw5* | Lower anterior chin | - Reshapes and projects the chin - Lifts the jawline - Improves submental skin excess |
| **Refinement** |  |  |
| Periorbital |  |  |
| Forehead *(F)*, 3-point forehead reshape | |  |
| *F1* | Medial forehead | - Reshapes the forehead - Improves forehead concavity - Indirectly improves the appearance of forehead lines - Indirectly lifts the brow |
| *F2* | Lateral forehead |  |
| *F3* | Central forehead |  |
| Lateral orbital *(O)*, 3-point lateral periorbital reshape^a^ | |  |
| *O1* | Central lateral orbital | - Improves the appearance of static lines and volume loss in the lateral periorbital area - Helps to reposition lateral canthus |
| *O2* | Lower lateral orbital |  |
| *O3* | Upper lateral orbital |  |
| Eyebrow *(E)*, 3-point eyebrow reshape | |  |
| *E1* | Eyebrow tail | - Lifts and projects the eyebrow - Indirectly improves the hollow eye appearance of the upper eyelid |
| *E2* | Eyebrow center |  |
| *E3* | Eyebrow head |  |
| Tear trough *(Tt)*, 3-point tear trough reshape^a^ | |  |
| *Tt1* | Central infraorbital | - Improves infraorbital area via the direct approach |
| *Tt2* | Lateral infraorbital |  |
| *Tt3* | Medial infraorbital |  |
| Glabella *(G)*, 2-point glabellar reshape | |  |
| *G1* | Lateral glabella | - Improves the appearance of static glabellar lines |
| *G2* | Central glabella | - Improves the appearance of cutaneous depressions |
| Perioral |  |  |
| Nasolabial fold *(NL)*, 3-point nasolabial reshape^b^ | |  |
| *NL1* | Upper nasolabial fold | - Reduces prominence of nasolabial folds via the direct approach |
| *NL2* | Central nasolabial fold |  |
| *NL3* | Lower nasolabial fold |  |
| Marionette line *(M)*, 3-point marionette reshape | |  |
| *M1* | Upper marionette line | - Improves the appearance of marionette lines |
| *M2* | Central marionette line |  |
| *M3* | Lower marionette line |  |
| Lip (Lp) |  |  |
| *Lp1* | Vermilion body | - Promotes lip augmentation - Promotes lip projection and eversion |
| *Lp^1^* | Upper lip |  |
| *Lp_1_* | Lower lip |  |
| *Lp2* | Cupid’s bow | - Gives structure to cupid’s bow area |
| *Lp3* | Lip border | - Gives structure to the white line/border - Indirectly decreases perioral lines |
| *Lp^3^* | Upper lip |  |
| *Lp_3_* | Lower lip |  |
| *Lp4* | Medial tubercle | - Provides projection or fullness of the medial tubercle of the upper lip |
| *Lp5* | Lateral tubercles | - Provides projection or fullness of the lateral tubercles of the lower lip |
| *Lp6* | Oral commissure | - Lifts and corrects the downturn of the corner of the mouth |
| *Lp7* | Philtrum column | - Gives structure and defines philtrum columns |
| *Lp8* | Perioral lines | - Corrects the perioral lines - Reshapes the cutaneous part of the lip |
| *Lp^8^* | Upper perioral lines |  |
| *Lp_8_* | Lower perioral lines |  |

TML, top-model look.

^a^Tear trough and orbital codes are reserved for specialists specifically trained in this technique and for those who have a sound knowledge of the anatomy and physiology of this particular area.

^b^Treatment of the nasolabial folds should start with assessment and treatment of the cheek (*Ck* codes), which may indirectly improve their appearance. Should this be insufficient, proceed to direct treatment using the *NLF* codes.

**SUPPLEMENTAL FIGURE LEGENDS**

**Figure S1.** Algorithm for deciding which MD Codes to treat (**a**) nasolabial folds, (**b**) small/recessed chin, (**c**) double chin, (**d**) lips, (**e**) perioral lines, and (**f**) marionette lines.

*C1,* labiomental angle; *C2,* chin apex; *C3,* anterior chin; *C4*, soft tissue pogonion; *C6,* prejowl sulcus; *Jw1*, mandible angle; *Jw2*, pre-auricular area; *Jw3*, mandible body; *Jw4*, lower prejowl; *Jw5*, lower anterior chin; *Lp1*, vermilion body (*Lp^1^* upper lip; *Lp_1_*, lower lip); *Lp2*, cupid’s bow; *Lp3*, lip border (*Lp^3^* upper; *Lp_3_* lower); *Lp4*, medial tubercle; *Lp5,* lateral tubercles; *Lp6*, oral commissure; *Lp7*, philtrum column; *Lp8*, perioral lines (*Lp^8^* upper; *Lp_8_* lower); *M1*, upper marionette line; *M2,* central marionette line; *M3*, lower marionette line; *NL1*, upper nasolabial fold; *NL2*, central nasolabial fold; *NL3*, lower nasolabial fold.

**Figure S2.** The components of the MD Codes are summarized in flash cards to communicate the technique rapidly in a digital application. Each card provides the associated product, layer, tool, delivery, and active number associated with a single MD code. For example, starting from the code and reading counterclockwise, this flash card (**a**) shows that for *Ck1*, the product is Voluma, injected at the supraperiosteal layer using a needle; the delivery is in a bolus; and the active number is 0.1 mL + 0.1 mL + 0.1 mL. A treatment plan with the formula for treating a tired look ([*Ck1 +Ck2 + Ck3] + [T1] + [Tt1 + Tt2 + Tt3]*) is communicated visually using the flashcards in panel (**b**).
