# Supplementary figures and images for: MD Codes™: A Methodological Approach to Facial Aesthetic Treatment with Injectable Hyaluronic Acid Fillers
Source: Aesthetic Plast Surg. 2020 May 22;45(2):690–709. doi: 10.1007/s00266-020-01762-7 (PMC8012343; doi:10.1007/s00266-020-01762-7)

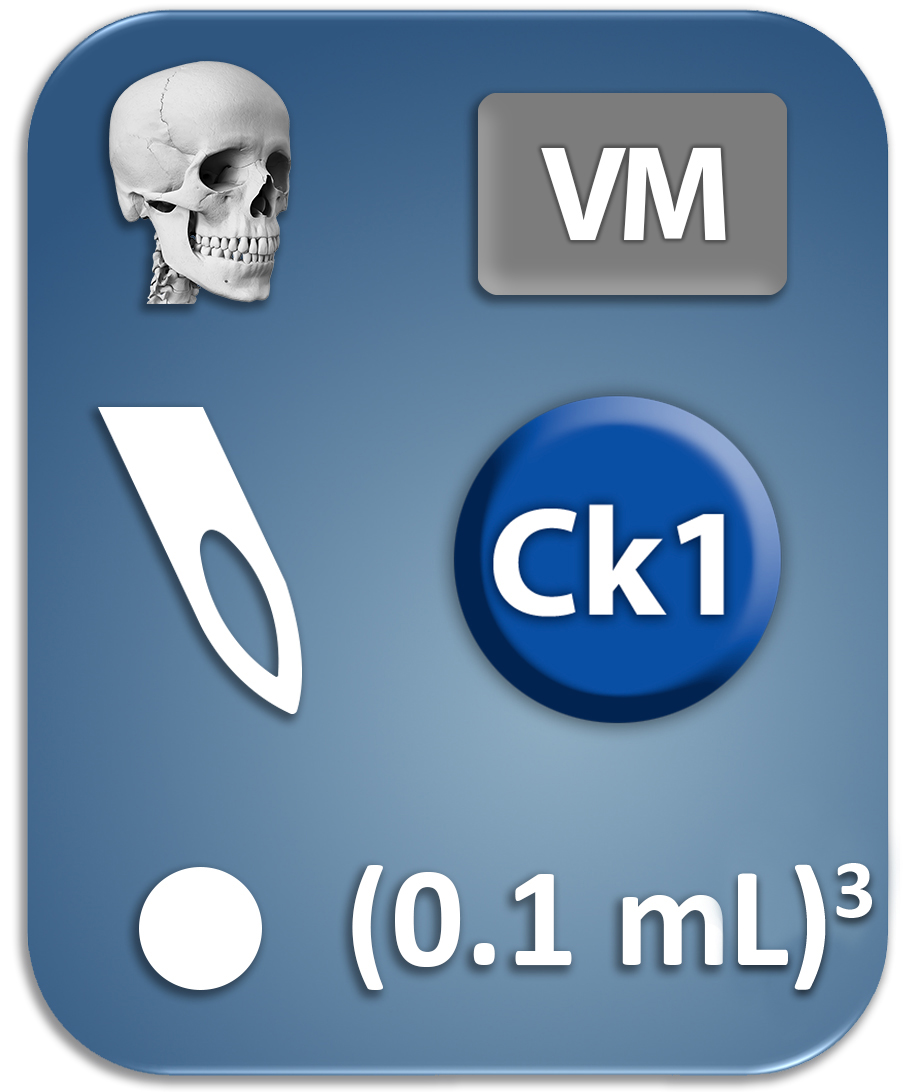

Supplement: Supplementary file 8 — Supplementary material 8 (TIFF 666 kb) [file 266_2020_1762_MOESM8_ESM.tif]

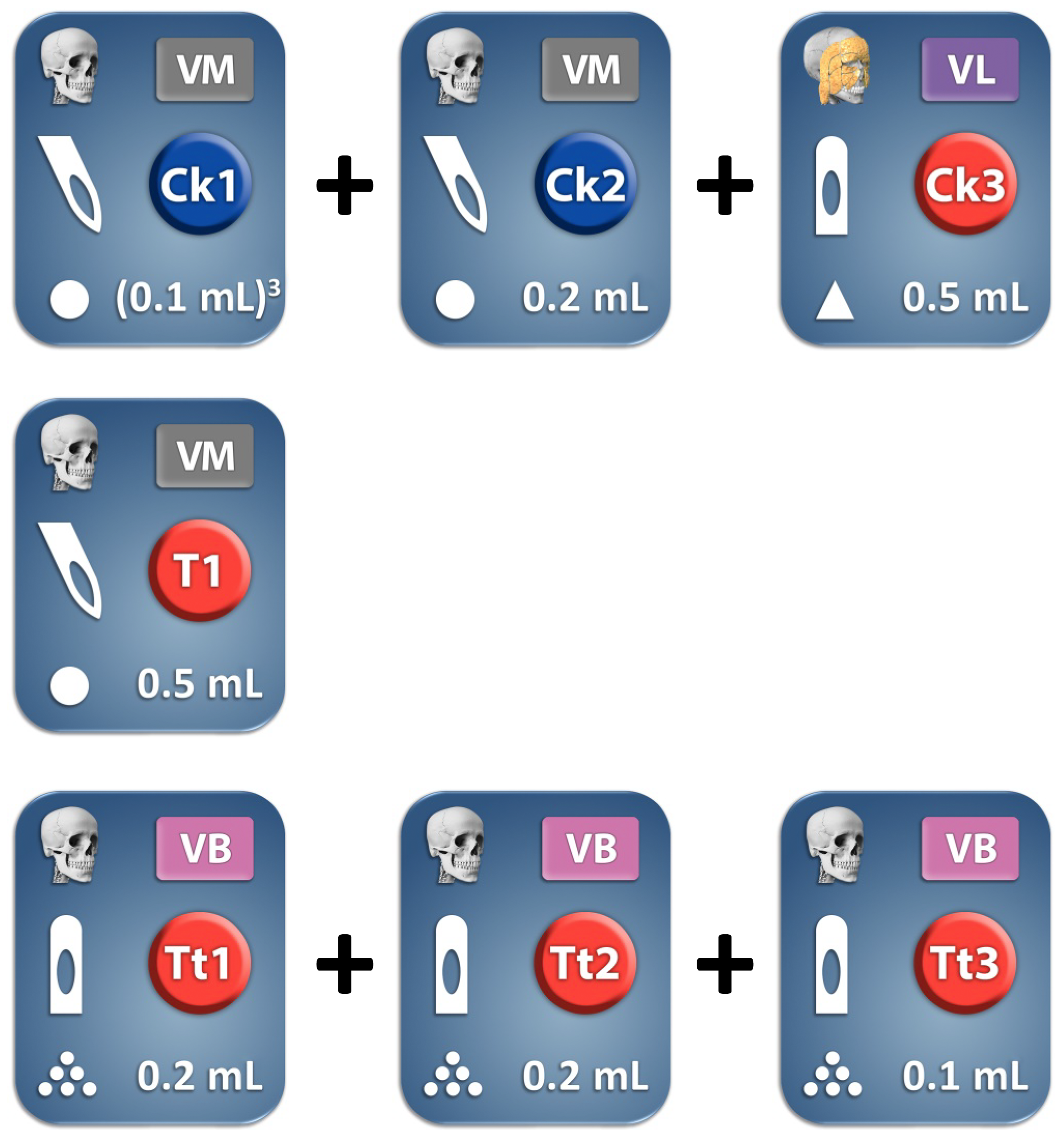

Supplement: Supplementary file 9 — Supplementary material 9 (TIFF 2858 kb) [file 266_2020_1762_MOESM9_ESM.tif]
